# Organic Fertilizer Substitution Regulates Nutrient Availability, Recovery, and Yield in Alpine Rapeseed (*Brassica napus* L.) Through Soil Enzyme Activity

**DOI:** 10.3390/plants15091302

**Published:** 2026-04-23

**Authors:** Runqi Quan, Jun Cao, Hejie Zhao, Jianguo Zhang, Wenyun Ding, Gensheng Chang, Xingxing Zhao, Jiaze Yu, Minjie Duan, Jinrui Zhou, Pinghui Liu, Danrui Liu, Wenxue Ba, Jun Wu

**Affiliations:** 1 College of Resources and Environmental Sciences, Gansu Agricultural University, Lanzhou 730070, China; quanrunqi0101@163.com (R.Q.); cgs0422@outlook.com (G.C.); 19119399235@163.com (X.Z.); yujz20020828@163.com (J.Y.); d2568312062@163.com (M.D.); 18828046865@163.com (J.Z.); wuhu2495@sina.com (P.L.); 17693601976@163.com (D.L.); bwxly@foxmail.com (W.B.); 2 Gannan Tibetan Autonomous Prefecture Academy of Agriculture, Forestry, Animal Husbandry and Grassland Sciences, Gannan 747000, China; cj18109411990@163.com (J.C.); 13321240991@163.com (H.Z.); 13893920820@163.com (J.Z.); 13909417997@163.com (W.D.)

**Keywords:** organic-chemical fertilizer substitution, nutrient availability, soil extracellular enzymes, nutrient-use efficiency

## Abstract

Livestock manure resources are abundant in the upper Yellow River basin on the eastern Tibetan Plateau, where rapeseed (*Brassica napus* L.) is grown under cold, short-season alpine conditions. To identify a suitable organic fertilizer substitution proportion, a two-year randomized complete block field experiment was conducted on Chestnut soil (Kastanozem) to compare mineral fertilization with 25%, 50%, 75%, and 100% replacement of mineral N by an organic fertilizer produced from composted cattle and sheep manure under equal total N, P, and K inputs. Grain yield was highest at 50% substitution, increasing by about 14% relative to mineral fertilization (*p* < 0.05), whereas 100% substitution slightly reduced yield. Increasing manure inputs enlarged soil organic carbon and total nutrient pools, but these increases were not accompanied by proportional increases in plant-available nutrients. Compared with mineral fertilization, 50% substitution increased available N, P, and K by about 18%, 34%, and 10%, respectively, and also increased the proportions of total N, P, and K present in available forms. Activities of the measured extracellular enzymes were generally 12–72% higher under 50% substitution than under mineral fertilization. A piecewise structural equation model indicated that yield improvement was associated mainly with greater nutrient uptake and recovery efficiency. Overall, moderate substitution best balanced nutrient accumulation, nutrient availability, efficiency, and productivity under the tested alpine conditions.

## 1. Introduction

The Tibetan Plateau functions as an important ecological security barrier for China [1]. On the eastern margin of the Tibetan Plateau, agricultural production is concentrated in the upper Yellow River headwaters, where fragile cultivated soils and a pronounced agro-pastoral mosaic create strong crop-livestock coupling and substantial opportunities for manure recycling [2]. In this alpine region, rapeseed (*Brassica napus* L.) is an important crop because of its relatively strong cold tolerance [3]. However, low temperature and a short growing season remain major constraints in the region, with mean annual temperatures of about 1–5.2 °C and frost-free periods as short as 64 days in high-altitude basins [4]. Organic fertilizer substitution is widely regarded as a promising strategy because it supplies nutrients, improves soil structure, and enhances microbial activity [5,6]. Its performance in high-altitude cold regions, however, is constrained because manure mineralization is strongly temperature-dependent [7]. Under cold conditions, nutrient release under full organic substitution may be too slow to meet crop demand during critical growth stages, potentially leading to yield penalties [8]. By contrast, partial substitution may stimulate microbial activity and organic matter turnover, thereby improving soil nutrient availability [9,10]. Whether this potential advantage can be realized under alpine conditions remains unclear.

Despite the documented benefits of organic substitution, the mechanisms linking substitution proportion with yield and nutrient-use efficiency in cold agroecosystems remain insufficiently understood [4]. First, most existing studies focus mainly on yield and basic soil properties, with less attention to the proportion of total nutrients present in available forms (nitrogen availability coefficient, NAC; phosphorus availability coefficient, PAC; and potassium availability coefficient, KAC), which may better reflect changes in in-season nutrient availability [11]. Second, although source-sink relationships are central to yield formation [12], relatively few studies have combined nutrient harvest index (NHI) and harvest index (HI) to evaluate nutrient translocation from vegetative organs to seeds under cold conditions [13,14]. Third, integrated assessments that jointly consider agronomic efficiency (AE), recovery efficiency (RE), and economic return remain limited [15,16]. A clearer understanding of these linked responses is needed to optimize fertilizer management. To address these issues, we evaluated whether organic fertilizer substitution could jointly improve yield, nutrient availability, and economic return. A two-year field experiment (2024–2025) was conducted at a representative site on the northeastern margin of the Qinghai-Tibet Plateau. This study links soil biological processes with plant nutrient translocation to explain yield formation in an alpine rapeseed system.

The objectives of this study were to: (1) determine how different organic fertilizer substitution proportions affect rapeseed yield and economic return on the eastern Tibetan Plateau; (2) quantify the responses of nutrient uptake and nutrient-use efficiency indicators (AE, RE, and NHI) to different substitution proportions; and (3) clarify the soil and plant processes associated with nutrient availability and source-sink coordination. We hypothesized that: (i) an intermediate substitution proportion would enhance enzyme-mediated nutrient availability more effectively than full organic substitution; and (ii) this improvement in nutrient availability, together with more efficient nutrient translocation to grain, would result in greater yield stability and nutrient-use efficiency than either sole mineral fertilization or full organic substitution.

## 2. Results

### 2.1. Soil Nutrient Availability and Enzymatic Drivers

#### 2.1.1. Soil Chemical Properties and Nutrients

**Figure 1:**
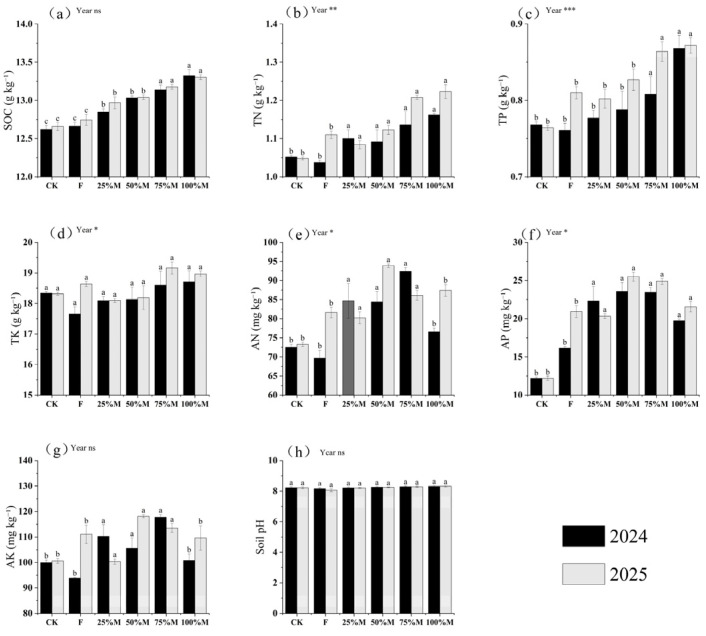
Soil chemical properties and nutrient status in the 0–20 cm layer under different organic substitution treatments in 2024 and 2025: (**a**) soil organic carbon (SOC), (**b**) total nitrogen (TN), (**c**) total phosphorus (TP), (**d**) total potassium (TK), (**e**) available nitrogen (AN), (**f**) available phosphorus (AP), (**g**) available potassium (AK), and (**h**) pH. Values are means ± SE (*n* = 3). Different lowercase letters indicate significant differences among treatments within each year according to Fisher’s LSD test at *p* < 0.05. *, **, and *** indicate significance at *p* < 0.05, *p* < 0.01, and *p* < 0.001, respectively; ns indicates not significant.

Across both seasons, manure substitution increased soil organic carbon (SOC) and total nutrient pools, with larger increases at higher substitution rates (Figure 1a–d). Relative to CK (no fertilizer), SOC increased by 3.0–3.3% under 50%M and by 4.1–5.6% under 75–100%M. Similar trends were observed for total nitrogen (TN) and total phosphorus (TP): TN under 75–100%M was 8.0–16.7% higher than CK, and TP under 100%M was 13.0–14.1% higher. By contrast, total potassium (TK) increased only slightly, by 2.0–3.5%.

Available nutrients showed a different pattern and were highest under partial rather than full substitution (Figure 1e–g). Compared with CK, 50%M increased available nitrogen (AN), available phosphorus (AP), and vailable potassium (AK) by 16.4–28.1%, 93.5–109.2%, and 5.7–17.4%, respectively; compared with F (100% mineral fertilizer), the corresponding increases were 15.0–21.1%, 21.8–46.0%, and 6.4–12.6%. Relative to 50%M, full substitution reduced AN, AP, and AK by 6.9–9.3%, 15.4–16.3%, and 4.6–7.2%, respectively. Overall, higher substitution rates favored nutrient accumulation, whereas partial substitution more effectively improved nutrient availability during the growing season.

#### 2.1.2. Proportions of Total Nutrients Present in Available Forms (NAC, PAC, and KAC)

**Figure 2:**
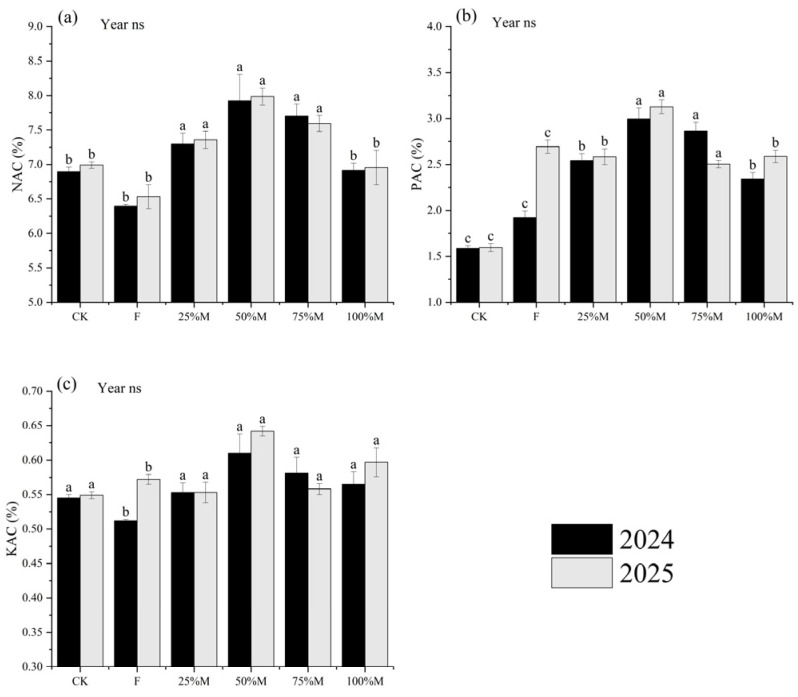
Indices describing the proportion of total nutrients present in available forms under different organic substitution treatments in 2024 and 2025: (**a**) nitrogen availability coefficient (NAC), (**b**) phosphorus availability coefficient (PAC), and (**c**) potassium availability coefficient (KAC). Values are means ± SE (*n* = 3). Different lowercase letters indicate significant differences among treatments within each year according to Fisher’s LSD test at *p* < 0.05. ns indicates not significant.

NAC, PAC, and KAC were highest under partial substitution, particularly under 50%M (Figure 2a–c). Relative to CK, 50%M increased NAC, PAC, and KAC by 14.2–14.9%, 89.0–95.9%, and 11.9–16.9%, respectively. Compared with F, the corresponding increases were 22.2–23.9%, 16.1–55.9%, and 12.2–19.1%.

By contrast, full substitution reduced these indices relative to 50%M. Under 100%M, NAC, PAC, and KAC were 12.7–12.9%, 17.2–21.8%, and 7.0–7.4% lower, respectively. These results indicate that partial substitution increased the share of total nutrients present in available forms, whereas high substitution enlarged total nutrient pools without a comparable increase in their available fractions.

#### 2.1.3. Soil Enzyme Activities

**Figure 3:**
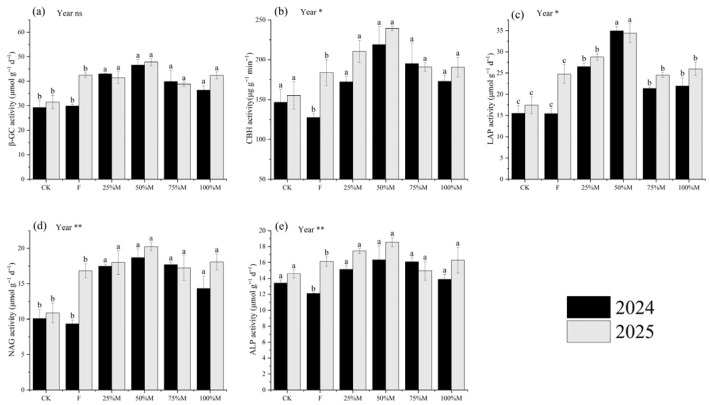
Soil extracellular enzyme activities under different organic substitution treatments in 2024 and 2025: (**a**) β-glucosidase (β-GC), (**b**) cellobiohydrolase (CBH), (**c**) leucine aminopeptidase (LAP), (**d**) N-acetyl-β-D-glucosaminidase (NAG), and (**e**) alkaline phosphatase (ALP). Values are means ± SE (*n* = 3). Different lowercase letters indicate significant differences among treatments within each year according to Fisher’s LSD test at *p* < 0.05. * and ** indicate significance at *p* < 0.05, *p* < 0.01, respectively. ns indicates not significant.

Extracellular enzyme activities, including β-glucosidase (β-GC), cellobiohydrolase (CBH), leucine aminopeptidase (LAP), N-acetyl-β-D-glucosaminidase (NAG), and alkaline phosphatase (ALP), were generally highest under partial substitution, especially under 50%M (Figure 3a–e). Compared with CK, 50%M increased β-GC by 51.7–59.0%, CBH by 49.5–54.3%, LAP by 97.3–125.2%, and NAG by 85.7–85.9%. Relative to F, 50%M increased β-GC, CBH, LAP, NAG, and ALP by 12.4–56.1%, 30.2–72.1%, 38.9–126.3%, 20.2–100.1%, and 15.1–34.8%, respectively. Under full substitution, enzyme activities were 10–37% lower than under 50%M, depending on the enzyme. Overall, partial substitution stimulated extracellular enzyme activity more effectively than full substitution.

### 2.2. Rapeseed Yield and Economic Benefits

**Figure 4:**
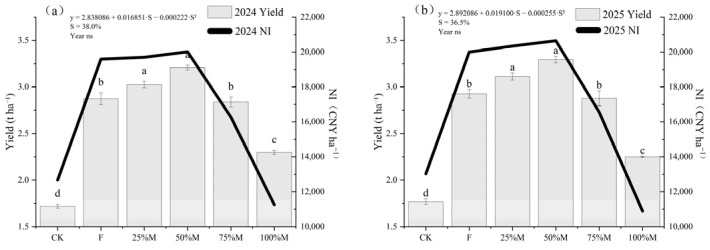
Rapeseed yield and net income (NI) under different organic substitution treatments in 2024 (**a**) and 2025 (**b**). Bars indicate yield (t ha^−1^) and lines indicate NI (Chinese yuan, CNY ha^−1^). Values are means ± SE (*n* = 3). Different lowercase letters indicate significant yield differences among treatments within each year (Fisher’s LSD, *p* < 0.05). ns indicates not significant.

Grain yield was highest under 50%M in both years (Figure 4a,b). Compared with F, 50%M increased yield by 14.2–14.5%, and compared with CK, by 81.6–82.3%. Yield under 75%M remained close to that under F, whereas yield under 100%M was 3.2–3.6% lower than under F.

Net income (NI) generally followed the same pattern as yield (Figure 4a,b), with the highest values under 50%M and similarly high values under 25%M. In contrast, economic return declined under 75%M and 100%M, particularly under full substitution. Quadratic regression excluding CK estimated a yield-maximizing substitution rate of 43–44%.

### 2.3. Yield Components and Resource Allocation

**Figure 5:**
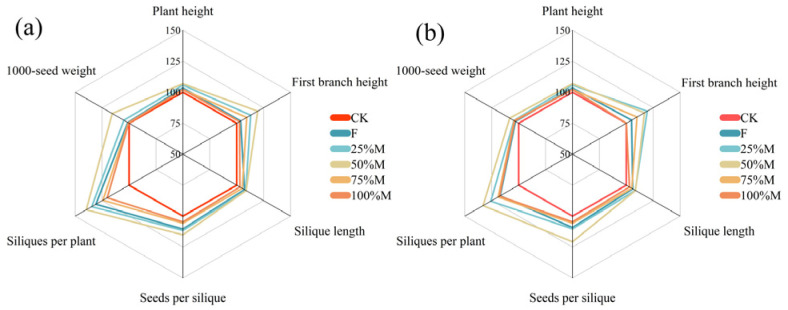
Radar plots of relative agronomic trait performance under different organic substitution treatments in 2024 (**a**) and 2025 (**b**). Traits include plant height, first branch height, silique length, seeds per silique, siliques per plant, and 1000-seed weight (CK set to 100 for each trait).

**Figure 6:**
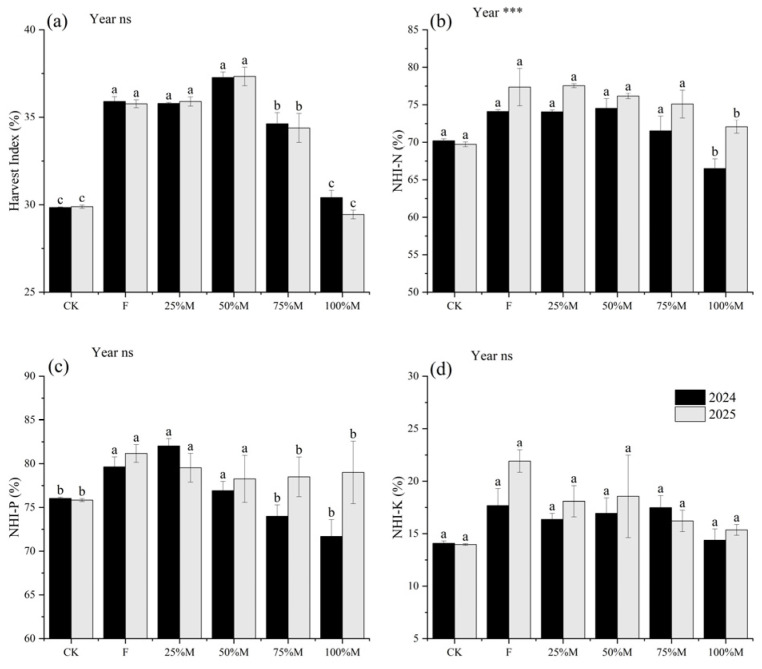
Harvest index and nutrient harvest indices under different organic substitution treatments in 2024 and 2025: (**a**) harvest index (HI), (**b**) nitrogen harvest index (NHI-N), (**c**) phosphorus harvest index (NHI-P), and (**d**) potassium harvest index (NHI-K). Values are means ± SE (*n* = 3). Different lowercase letters indicate significant differences among treatments within each year according to Fisher’s LSD test at *p* < 0.05. *** and ns indicate significance at *p* < 0.001 and not significant, respectively.

Yield responses were accompanied by coordinated changes in agronomic traits and reproductive allocation (Figure 5a,b and Figure 6a–d). Partial substitution generally improved the overall agronomic trait profile relative to CK, with the largest overall improvement under 50%M in both years (Figure 5a,b). The clearest advantages of 50%M were observed for first branch height, seeds per silique, and 1000-seed weight, whereas silique length varied little among treatments. These advantages weakened under 75%M and 100%M, although the overall treatment pattern was broadly similar between years.

Harvest index (HI) remained relatively high under partial substitution but declined at higher substitution rates (Figure 6a). Compared with 75%M and 100%M, HI under 50%M was higher by 2.65–2.95 and 6.86–7.89 percentage points, respectively. Nutrient harvest indices showed element-specific responses (Figure 6b–d). NHI-N and NHI-K under 100%M were 4.10–8.02 and 2.56–3.20 percentage points lower than under 50%M, respectively, whereas differences in NHI-P were smaller and less consistent. Overall, higher substitution rates reduced seed partitioning relative to partial substitution.

### 2.4. Organ-Specific N, P, and K Accumulation at Maturity

**Figure 7:**
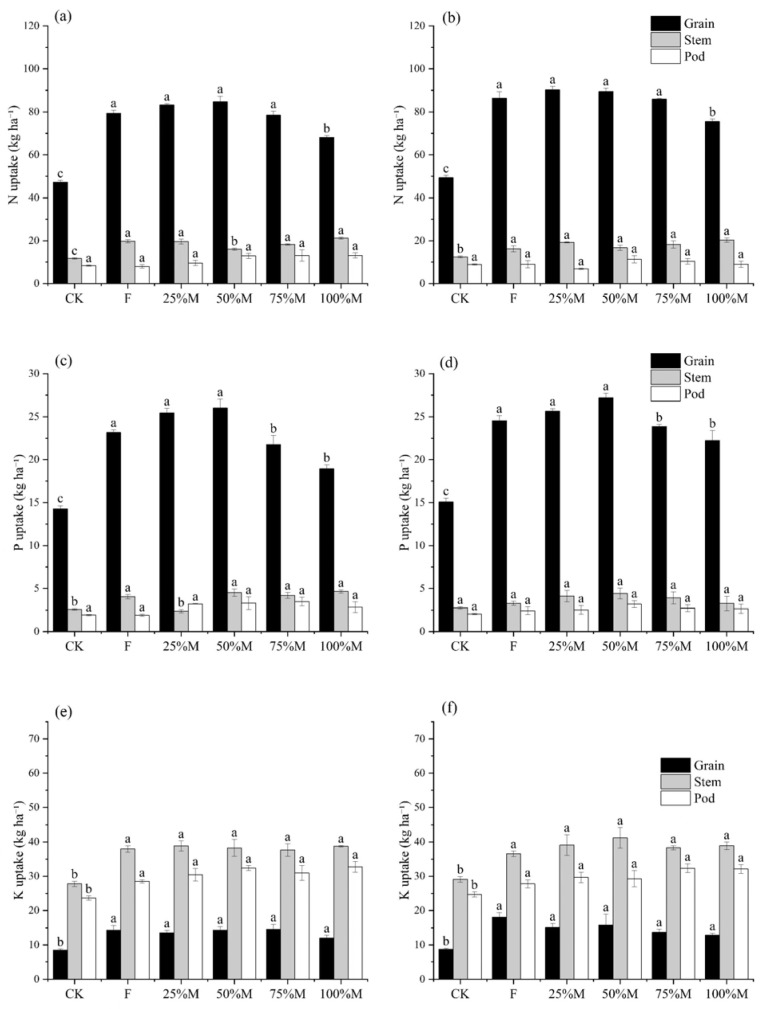
Organ-specific accumulation of nitrogen (N), phosphorus (P), and potassium (K) in rapeseed at maturity under different organic substitution treatments in 2024 and 2025: (**a**) N in 2024, (**b**) N in 2025, (**c**) P in 2024, (**d**) P in 2025, (**e**) K in 2024, and (**f**) K in 2025. Values are means ± SE (*n* = 3). Different lowercase letters indicate significant differences among treatments within each year according to Fisher’s LSD test at *p* < 0.05. Nutrient accumulation (kg ha^−1^) was calculated as organ dry biomass × nutrient concentration.

Organ-specific nutrient accumulation indicated that the yield advantage under 50%M was mainly associated with greater nutrient allocation to grain (Figure 7a–f). Relative to F, 50%M increased grain N and grain P accumulation by 3.6–6.7% and 10.7–12.3%, respectively. Relative to 100%M, grain N, grain P, and grain K accumulation under 50%M were higher by 18.5–24.4%, 22.3–37.5%, and 19.7–23.2%, respectively. For grain K, the comparison between 50%M and F differed between years, but 50%M remained higher than 100%M in both seasons (Figure 7e,f).

By contrast, nutrient retention in stems and pods did not follow the same pattern as that in grain. Stem N tended to be higher under high substitution than under 50%M, indicating greater nutrient retention in non-harvested tissues at high substitution rates. Responses in stems and pods varied among nutrients, suggesting that the main treatment effect was not a uniform increase in nutrient accumulation across all organs, but a greater transfer of nutrients to grain under partial substitution.

### 2.5. Fertilizer Use Efficiency

**Figure 8:**
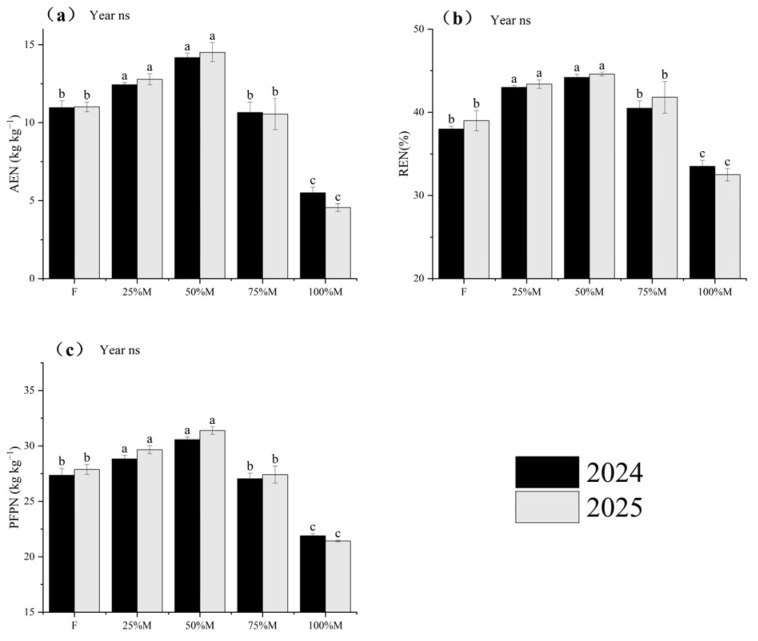
Nitrogen use efficiency indices under different organic substitution treatments in 2024 and 2025: (**a**) agronomic efficiency of N (AEN), (**b**) recovery efficiency of N (REN), and (**c**) partial factor productivity of N (PFPN). Values are means ± SE (*n* = 3). Different lowercase letters indicate significant differences among treatments within each year according to Fisher’s LSD test at *p* < 0.05. ns indicates not significant.

**Figure 9:**
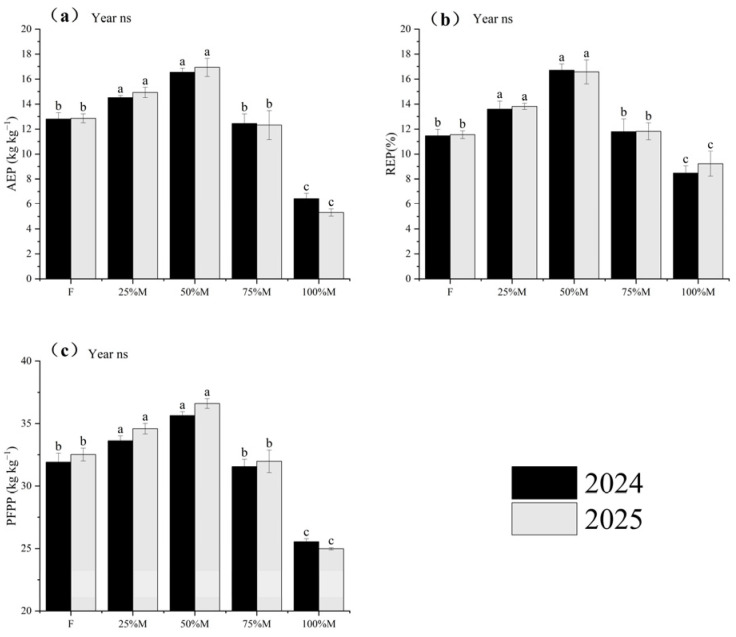
Phosphorus use efficiency indices under different organic substitution treatments in 2024 and 2025: (**a**) agronomic efficiency of P (AEP), (**b**) recovery efficiency of P (REP), and (**c**) partial factor productivity of P (PFPP). Values are means ± SE (*n* = 3). Different lowercase letters indicate significant differences among treatments within each year according to Fisher’s LSD test at *p* < 0.05. ns indicates not significant.

**Figure 10:**
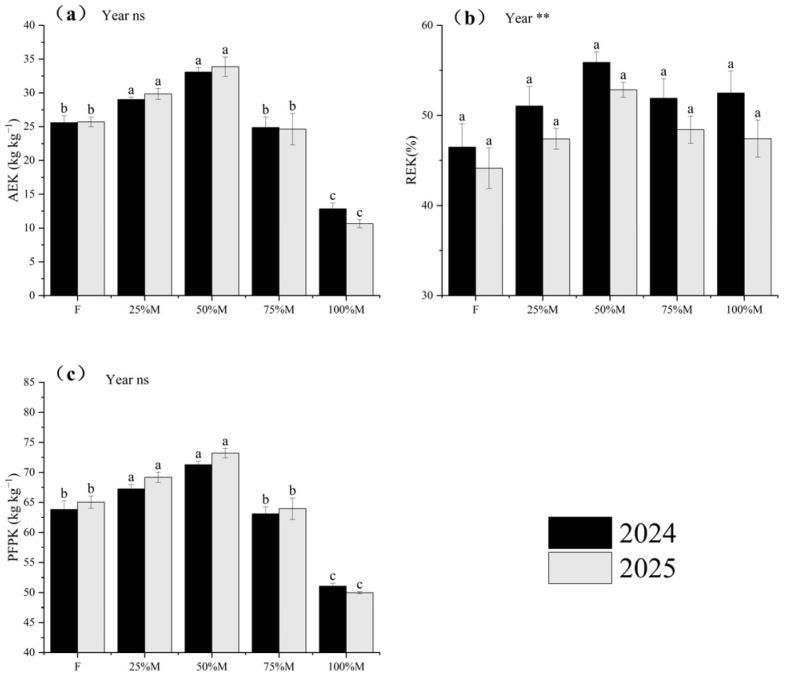
Potassium use efficiency indices under different organic substitution treatments in 2024 and 2025: (**a**) agronomic efficiency of K (AEK), (**b**) recovery efficiency of K (REK), and (**c**) partial factor productivity of K (PFPK). Values are means ± SE (*n* = 3). Different lowercase letters indicate significant differences among treatments within each year according to Fisher’s LSD test at *p* < 0.05. ** and ns indicate significance at *p* < 0.01 and not significant, respectively.

Fertilizer-use efficiency indices agronomic efficiency (AE), recovery efficiency (RE), and partial factor productivity (PFP) responded clearly to fertilizer source structure, and the overall treatment pattern was similar across N, P, and K (Figure 8, Figure 9 and Figure 10). Across nutrients, 50%M generally produced the highest efficiency values, followed by 25%M, whereas 75%M was often close to F and 100%M consistently showed the lowest values. In general, treatments with higher AE and PFP also maintained higher RE, indicating that the yield advantage under partial substitution was accompanied by improved nutrient recovery.

#### 2.5.1. Nitrogen Use Efficiency (NUE)

Nitrogen use efficiency (NUE) was highest under partial substitution, particularly under 50%M (Figure 8a–c). Relative to F, 50%M increased AEN, REN, and PFPN by 29.2–31.8%, 14.4–16.3%, and 11.7–12.5%, respectively. By contrast, full substitution reduced NUE relative to 50%M. Under 100%M, AEN, REN, and PFPN were 61.2–68.6%, 24.2–27.1%, and 28.4–31.7% lower, respectively. These results indicate that moderate substitution improved both N recovery and yield return per unit N input.

#### 2.5.2. Phosphorus Use Efficiency (PUE)

Phosphorus use efficiency (PUE) showed a similar response pattern (Figure 9a–c). Compared with F, 50%M increased AEP, REP, and PFPP by 29.2–31.7%, 43.5–45.7%, and 11.7–12.5%, respectively. Relative to 50%M, full substitution markedly reduced P efficiency, with AEP, REP, and PFPP decreasing by 61.2–68.6%, 44.4–49.3%, and 28.4–31.7%, respectively. Thus, partial substitution improved both P recovery and productivity per unit P input.

#### 2.5.3. Potassium Use Efficiency (KUE)

Potassium use efficiency (KUE) followed the same overall ranking, although the response of K recovery was smaller than that of N and P recovery (Figure 10a–c). Relative to F, 50%M increased AEK, REK, and PFPK by 29.2–31.7%, 19.7–20.2%, and 11.7–12.5%, respectively. Compared with 50%M, 100%M reduced AEK and PFPK by 61.2–68.6% and 28.4–31.7%, respectively, whereas the decline in REK was smaller, at 6.1–10.3%. Overall, the advantage of partial substitution for K use was expressed mainly through higher productivity per unit input, whereas K recovery was comparatively less sensitive.

Across N, P, and K, 50%M showed the strongest overall efficiency performance, whereas 25%M also maintained relatively high values (Figure 8, Figure 9 and Figure 10). AE and PFP for all three nutrients were highest under 50%M, while RE also peaked under 50%M for N and P. For K, RE remained relatively high across the 25–75%M range. Overall, moderate substitution improved fertilizer-use efficiency more effectively than full substitution.

### 2.6. Piecewise SEM Links Fertilizer Source Structure to Enzyme Activity, Nutrient Availability, Recovery Efficiency, and Yield

**Figure 11:**
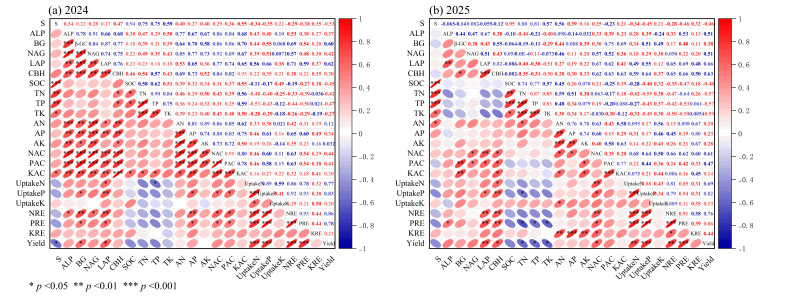
Season-specific Pearson correlation heatmaps among organic substitution rate (S), the Enzyme index, Soil Pool index, Available index, Uptake index, recovery efficiency index (RE), and yield in fertilized plots: (**a**) 2024 and (**b**) 2025. The Enzyme index was calculated from β-glucosidase (β-GC), cellobiohydrolase (CBH), leucine aminopeptidase (LAP), N-acetyl-β-D-glucosaminidase (NAG), and alkaline phosphatase (ALP); the Soil Pool index from soil organic carbon (SOC), total nitrogen (TN), total phosphorus (TP), and total potassium (TK); the Available index from available nitrogen (AN), available phosphorus (AP), and available potassium (AK); the Uptake index from plant N, P, and K uptake; and RE from N, P, and K recovery efficiency. Correlations were calculated using fertilized plots only (n = 15 per year; five fertilized treatments × three replicates); CK was excluded because S is not defined for the unfertilized control. Upper triangles show Pearson correlation coefficients (r), and lower triangles show correlation direction and magnitude.

Season-specific Pearson correlation heatmaps showed coordinated relationships among substitution rate, soil nutrient pools, enzyme activity, nutrient availability, nutrient uptake, recovery efficiency, and yield (Figure 11a,b). Positive correlations were strongest among nutrient availability, uptake, recovery efficiency, and yield, whereas soil nutrient pools were less closely associated with these downstream variables.

**Figure 12:**
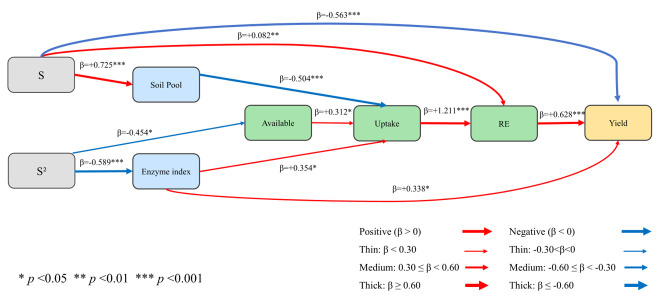
Non-saturated piecewise structural equation model (SEM) linking organic substitution rate (S) and its quadratic term (S^2^), the Soil Pool index, Enzyme index, Available index, Uptake index, recovery efficiency index (RE), and yield in fertilized plot-year observations. The Soil Pool index was calculated from soil organic carbon (SOC), total nitrogen (TN), total phosphorus (TP), and total potassium (TK); the Enzyme index from β-glucosidase (β-GC), cellobiohydrolase (CBH), leucine aminopeptidase (LAP), N-acetyl-β-D-glucosaminidase (NAG), and alkaline phosphatase (ALP); the Available index from available nitrogen (AN), available phosphorus (AP), and available potassium (AK); the Uptake index from plant N, P, and K uptake; and RE from N, P, and K recovery efficiency. Values beside arrows are standardized path coefficients (β). Model fit: Fisher’s C = 8.226, df = 12, *p* = 0.767.

To integrate these relationships, a non-saturated piecewise structural equation model (SEM) was fitted using fertilized plot-year observations (Figure 12). Model fit was acceptable (Fisher’s C = 8.226, df = 12, *p* = 0.767). Because all variables were standardized before model fitting, the path coefficients (β) are directly comparable, and larger absolute β values indicate stronger associations.

Substitution increased Soil Pool status (β = 0.725, *p* < 0.001). Uptake was positively associated with Available (β = 0.312, *p* = 0.019) and the Enzyme index (β = 0.354, *p* = 0.014), but negatively associated with Soil Pool status (β = −0.504, *p* < 0.001). Recovery efficiency was mainly explained by Uptake (β = 1.211, *p* < 0.001) and was also positively associated with substitution (β = 0.082, *p* = 0.002). Yield was positively associated with recovery efficiency (β = 0.628, *p* < 0.001) and the Enzyme index (β = 0.338, *p* = 0.014), but negatively associated with substitution (β = −0.563, *p* < 0.001). Overall, yield was more closely linked to enzyme-mediated nutrient availability, uptake, and recovery than to expansion of total soil nutrient pools alone.

Overall, treatment responses were broadly similar across the two seasons, although some soil nutrient, enzyme, uptake, yield, and economic indicators showed significant Year × Treatment interactions.

## 3. Discussion

### 3.1. Soil Nutrient Pools, Nutrient Availability, and Enzyme-Mediated Nutrient Release Under Moderate Organic Substitution

Across the two growing seasons, manure substitution led to a clear divergence between soil nutrient accumulation and in-season nutrient availability. Higher substitution proportions, especially 75%M and 100%M, increased soil organic carbon and total nutrient contents more strongly, indicating that greater organic inputs favored soil C sequestration and nutrient storage [17,18]. In contrast, the highest available N, P, and K contents, together with the highest proportions of total nutrients present in available forms, were observed under 50%M rather than 100%M. Similar patterns have been reported in recent studies in which partial organic substitution outperformed either sole mineral fertilization or excessive manure replacement in improving soil quality and sustaining crop productivity [19,20].

This pattern suggests that high substitution favored nutrient pool accumulation, but not necessarily nutrient release during the growing season. The conversion of manure-derived nutrients into plant-available forms depends on microbial decomposition and extracellular enzyme activity, which are regulated by temperature, soil properties, and substrate quality [21,22]. Under alpine cold conditions, low temperature can slow mineralization and delay nutrient release, increasing the likelihood that nutrients remain in organic or less available forms during periods of high crop demand [21,23]. This may explain why 100%M increased SOC and total nutrient pools but did not maintain available nutrient levels comparable to those under 50%M. Moreover, the agronomic effects of manure substitution vary with manure properties, substitution proportion, climate, and soil type, indicating that the superiority of a given substitution proportion is strongly context dependent [24,25].

The enzyme responses further support the advantage of moderate substitution rates. β-glucosidase and cellobiohydrolase are involved in cellulose degradation, N-acetyl-β-D-glucosaminidase and leucine aminopeptidase contribute to organic N depolymerization, and alkaline phosphatase is important for organic P hydrolysis in alkaline soils [21,22]. In the present study, 50%M maintained higher β-GC, CBH, LAP, NAG, and ALP activities than both F and 100%M, indicating a stronger biochemical capacity for coordinated C, N, and P turnover. These results suggest that moderate substitution provided a more favorable balance among nutrient input, microbial processing, and nutrient release than full substitution.

Taken together, the advantage of 50%M reflects not only higher soil available nutrient contents, but also a more balanced soil functional response. Although 25%M and 50%M showed similar short-term yield and economic performance, 50%M exhibited clearer and more consistent advantages in available nutrients, nutrient availability proportions, and extracellular enzyme activities. Under the tested alpine rapeseed conditions, 50%M therefore appears to provide a more suitable balance between short-term nutrient supply and progressive soil fertility improvement than either lower substitution or full replacement of mineral fertilizer.

### 3.2. Plant Nutrient Uptake and Reproductive Allocation Responses to Moderate Substitution

Moderate organic substitution improved yield formation mainly by enhancing nutrient acquisition and grain transfer. Recent studies have shown that partial replacement of mineral fertilizer with organic inputs can sustain crop productivity by improving nutrient uptake and nutrient-use efficiency, whereas excessive substitution may weaken the synchrony between nutrient release and crop demand [19,26,27]. In oilseed rape, seed yield is highly responsive to nutrient status because N supply regulates branch development, silique formation, seed filling, and final seed yield [28,29].

In the present study, 50%M increased grain N, P, and K accumulation, whereas higher substitution tended to retain a larger proportion of nutrients in stems and pods. This indicates that the advantage of moderate substitution lies not simply in greater total nutrient uptake, but in more effective partitioning of acquired nutrients to reproductive organs. Oilseed rape is characterized by incomplete nutrient remobilization, and post-flowering yield formation depends on the coordination of continued nutrient uptake, leaf and silique photosynthesis, and senescence-associated remobilization [30,31]. Maintaining nutrient supply during the reproductive stage is therefore essential for sustaining grain filling and improving harvest-related allocation [28,31,32].

The difference between 25%M and 50%M is therefore better interpreted in terms of physiological coordination rather than short-term yield alone. Although the two treatments produced similar short-term yield and economic returns, 50%M showed clearer advantages in grain nutrient accumulation and harvest-related allocation, indicating stronger coupling between nutrient uptake and reproductive partitioning to seed. In rapeseed, harvest index reflects the integrated outcome of biomass production, source-sink balance, and seed allocation, rather than biomass accumulation alone [32,33].

Direct comparison with other studies should nevertheless be made cautiously, because plant nutrient uptake and partitioning responses to organic substitution are influenced by soil type, manure source, substitution proportion, fertilizer management, and overall cropping conditions [19,27,34]. Under the tested alpine rapeseed conditions, 50%M therefore appears to provide a more suitable balance between nutrient acquisition and transfer to the harvested seed than either lower substitution or full replacement of mineral fertilizer.

### 3.3. Nutrient-Use Efficiency and SEM-Based Mechanistic Integration

Moderate organic substitution improved fertilizer-use efficiency mainly by enhancing nutrient capture and the conversion of nutrient inputs into harvested yield. Recent studies have shown that partial replacement of mineral fertilizer with organic inputs can maintain crop productivity while increasing agronomic or recovery efficiency, although the magnitude of this benefit remains strongly dependent on nutrient management, soil conditions, and cropping context [16,35,36]. In rapeseed, improving nutrient-use efficiency is particularly important because seed yield is highly responsive to N supply, but excessive N input often reduces efficiency without producing proportional yield gains [27,37].

In the present study, 50%M consistently achieved higher AE, RE, and PFP than F and 100%M, indicating that moderate substitution improved both nutrient recovery and yield return per unit input. This suggests that the advantage of 50%M depended less on total nutrient input than on the synchrony among nutrient release, plant uptake, and reproductive demand. At high substitution rates, nutrient release from organic sources was likely less synchronized with crop demand, thereby reducing in-season recovery and weakening the conversion of absorbed nutrients into grain yield. Similar studies have shown that organic substitution improves nutrient supply efficiency only when nutrient release is effectively coordinated with crop uptake and soil biological activity [35,38].

The SEM further clarified this pattern, showing that yield improvement was transmitted mainly through the pathway from nutrient availability to plant uptake and then to recovery efficiency, rather than through expansion of soil nutrient pools alone. This interpretation is consistent with recent studies showing that the agronomic benefits of organic inputs are often mediated by changes in nutrient availability, nutrient uptake, and fertilizer-use efficiency, whereas the treatment effect itself is largely indirect [35,39]. Within this framework, the standardized path coefficients indicate the relative strength of linked processes, and the acceptable global fit suggests that the proposed pathway is broadly supported by the observed data.

The difference between 25%M and 50%M is therefore better interpreted as one of system efficiency rather than short-term yield alone. Although the two treatments produced similar short-term yield and economic returns, 50%M showed a more coordinated availability-uptake-recovery-yield relationship, providing a stronger mechanistic basis for identifying it as the more suitable substitution proportion under the tested alpine rapeseed conditions. Cross-study extrapolation should nevertheless be made cautiously, because field and global analyses consistently show that nutrient-use efficiency responses to organic substitution are strongly conditioned by site and management context [25,36].

### 3.4. Limitations and Implications for Future Research

The present study should be interpreted as a short-term field evaluation of early cumulative responses rather than as a long-term assessment of system equilibrium. Although repeated application of the same treatments to the same plots over two growing seasons allowed robust treatment differences to be identified, longer-term studies have shown that organic fertilizer substitution can continue to modify soil properties, productivity, and system sustainability over much longer time scales [38,40]. Accordingly, the current results support a suitable substitution proportion under the tested conditions, but they should not be directly extrapolated to long-term system behavior.

A second limitation is the narrow scope of inference. This experiment was conducted in an alpine cold-humid rapeseed system on alkaline Kastanozem using one specific source of composted cattle and sheep manure. However, the agronomic effects of organic fertilizer substitution vary with fertilizer type, compost maturity, nutrient composition, soil properties, and climate, indicating that the superiority of a given substitution proportion is strongly context dependent [25,41,42]. In addition, organically bound nutrients in composted manures may be released more slowly than those from mineral fertilizers, which can further alter the synchrony between nutrient supply and crop demand under cold conditions [25,41].

The equal annual N-P-K input design standardized total nutrient supply across fertilized treatments, but it did not eliminate differences in seasonal nutrient release, interannual carryover, or residual nutrient accumulation. This issue is particularly important for phosphorus, because manure-derived P may persist in soil beyond the year of application and contribute to residual or legacy effects over longer periods [39,43]. Accordingly, the medium- and long-term consequences of repeated organic fertilizer substitution cannot be fully resolved in a two-year experiment.

Future research should therefore combine longer-term, multi-site field experiments with direct measurements of nutrient mineralization, seasonal nutrient dynamics, and residual effects across contrasting manure sources and soils. Integrating these measurements with rhizosphere processes, microbial indicators, and environmental loss pathways would help determine whether the advantage of moderate substitution remains stable under prolonged management and across broader agroecological conditions [25,40,42].

## 4. Materials and Methods

### 4.1. Site Description

**Figure 13:**
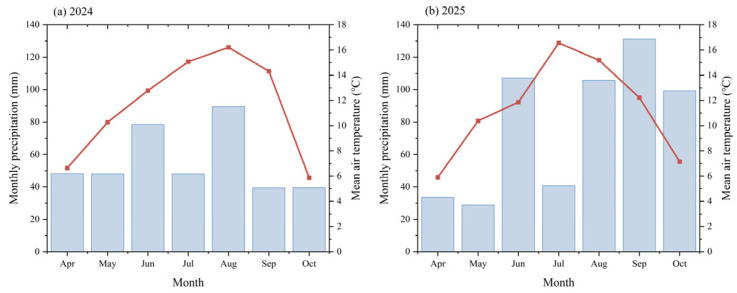
Monthly precipitation and mean air temperature during the rapeseed growing seasons (April–October) in (**a**) 2024 and (**b**) 2025 at the meteorological station nearest to the experimental site. Bars indicate monthly precipitation, and the line indicates monthly mean air temperature.

The field experiment was conducted over two consecutive growing seasons (2024–2025) at a research station on the northeastern margin of the Tibetan Plateau (103°20′21″ E, 34°44′35″ N) at an elevation of approximately 2800 m. The site is representative of alpine cold-humid agroecosystems in the eastern Tibetan Plateau. The region has a typical plateau continental monsoon climate, with a mean annual temperature of 3.2 °C, a frost-free period of approximately 64 days, and a long-term mean annual precipitation of 490 mm. The soil at the experimental site is classified as Chestnut soil (Kastanozem), which is the dominant soil type in this ecological zone. Meteorological data for the rapeseed growing seasons (April–October) in 2024 and 2025, including monthly mean air temperature and precipitation, were obtained from the meteorological station nearest to the experimental site and are shown in Figure 13. To determine the initial soil fertility status, topsoil (0–20 cm) was sampled before sowing in 2024, and the baseline physicochemical properties are presented in Table 1.

### 4.2. Experimental Design and Treatments

The field experiment was conducted in 2024 and 2025 using a randomized complete block design with six treatments and three replicates, giving a total of 18 plots. Each plot measured 6 m × 4 m (24 m^2^). The treatments were CK (no fertilizer), F (100% mineral fertilizer), and 25%M, 50%M, 75%M, and 100%M, representing 25%, 50%, 75%, and 100% replacement of mineral N with organic fertilizer N, respectively. All fertilized treatments were established under an equal total N-P-K input principle. Accordingly, total inputs of N, P_2_O_5_, and K_2_O were kept constant across fertilized treatments, whereas the N source gradually shifted from mineral fertilizer to organic fertilizer. Mineral fertilizers included urea (46% N), superphosphate (12% P_2_O_5_), and potassium sulfate (52% K_2_O). Detailed fertilizer application rates are shown in Table 2.

The organic fertilizer used in this study was a commercial product (“Tandifeng”) manufactured by Lintan Hongzheng Organic Fertilizer Co., Ltd. (Lintan, China). It was produced from composted cattle and sheep manure and contained 38.71% organic matter, 1.92% N, 1.61% P_2_O_5_, and 0.69% K_2_O. Its moisture content was 18.9%, and its C:N ratio was 11.7.

All fertilizers were applied once before sowing as basal fertilizer, and no topdressing was applied during the growing season. Mineral and organic fertilizers were uniformly broadcast and incorporated into the soil before sowing. Fertilizer rates in Table 2 are expressed on a dry-matter basis. The same treatments were re-applied to the same plots in the second year to assess short-term cumulative effects under equal annual total N, P, and K inputs. Rapeseed (*Brassica napus* L.) cv. ‘Qingza No. 7’ was sown in April and harvested in October in both years. No irrigation was applied during the growing season. Weeds were removed manually as required, and pest and disease management followed local production practices and was kept uniform across all plots.

### 4.3. Sample Collection and Preparation

Plant and soil samples were collected at harvest maturity (Biologische Bundesanstalt, Bundessortenamt and CHemical industry growth scale, BBCH 89) in October 2024 and 2025. In each plot, 10 plants were randomly sampled from the central rows, excluding border plants to avoid edge effects. These plants were separated into grain, stem, and pod fractions for biomass and nutrient analyses. The remaining plants in each plot were harvested to determine grain yield.

Plant samples were first oven-dried at 105 °C for 30 min and then at 70 °C to constant weight. Dry weight was recorded for biomass determination. The dried samples were ground to pass through a 0.5 mm sieve and stored for analysis of N, P, and K concentrations.

After plant harvest, soil samples were collected from the 0–20 cm plow layer of each plot using a five-point diagonal sampling method and combined into one composite sample per plot. After transport to the laboratory, each sample was thoroughly homogenized and divided into two subsamples. One subsample was kept field-moist and stored at 4 °C for extracellular enzyme assays. The other was air-dried, gently crushed, and sieved for chemical analyses. Soil passing through a 2 mm sieve was used for pH and available nutrient determinations, whereas soil passing through a 0.149 mm sieve was used for soil organic carbon and total nutrient analyses.

### 4.4. Soil and Plant Chemical Analysis

Soil pH was measured in a 1:2.5 (*w*/*v*) soil-to-water suspension using a pH meter. Soil organic carbon was determined by the potassium dichromate oxidation-external heating method. Total nitrogen was determined by the Kjeldahl method using an automatic Kjeldahl nitrogen analyzer (K9840, Hanon Instruments Co., Ltd., Jinan, China) after H_2_SO_4_ digestion with a catalyst. Total phosphorus was determined colorimetrically after H_2_SO_4_-HClO_4_ digestion using a UV-Vis spectrophotometer (UV-2100, UNICO Instrument Co., Ltd., Shanghai, China). Total potassium was measured by flame photometry using a flame photometer (FP6410, Shanghai Xinyi Instrument Co., Ltd., Shanghai, China) after NaOH fusion. Available nitrogen was determined by the alkali-hydrolyzable diffusion method. Available phosphorus was extracted with 0.5 mol L^−1^ NaHCO_3_ (pH 8.5) and determined colorimetrically using the UV-Vis spectrophotometer. Available potassium was extracted with 1.0 mol L^−1^ NH_4_OAc and measured by flame photometry using the flame photometer [44,45,46].

Plant samples were digested with H_2_SO_4_-H_2_O_2_. Plant N concentration was determined by the Kjeldahl method using the automatic Kjeldahl nitrogen analyzer, plant P concentration was determined colorimetrically using the UV–Vis spectrophotometer, and plant K concentration was measured by flame photometry using the flame photometer [47].

### 4.5. Soil Extracellular Enzyme Activities

Activities of five extracellular enzymes involved in soil C, N, and P acquisition were measured in field-moist soil collected from the 0–20 cm layer at harvest using a 96-well microplate fluorometric method following Saiya-Cork et al. [48]. β-glucosidase (β-GC) and cellobiohydrolase (CBH) were assayed as C-acquiring enzymes, leucine aminopeptidase (LAP) and N-acetyl-β-D-glucosaminidase (NAG) as N-acquiring enzymes, and alkaline phosphatase (ALP) as a P-acquiring enzyme. These enzymes represent key steps in the depolymerization of cellulose, proteins, chitin, and organic phosphorus and therefore reflect the potential for soil C, N, and P acquisition [48]. Because the experimental soil was alkaline, ALP was measured instead of acid phosphatase [49]. Briefly, 0.02 g of field-moist soil was suspended in 50 mL of buffer and stirred for approximately 30 min. A 70 μL aliquot of the supernatant was then transferred to a 96-well microplate, and 130 μL of the corresponding fluorogenic substrate solution was added to each well. After incubation under controlled conditions, the reaction was terminated with 10 μL of 1.0 M NaOH, and fluorescence was measured using a microplate reader (Tecan Spark, Tecan Group Ltd., Zurich, Switzerland). Enzyme activities were expressed on an oven-dry soil basis.

### 4.6. Calculations

To comprehensively evaluate the performance of organic substitution strategies, physiological, nutritional, soil, and economic indices were calculated using data from the 2024 and 2025 growing seasons. To assess the efficiency of biomass partitioning and nutrient translocation from vegetative to reproductive organs, harvest index (HI) and nutrient harvest index (NHI) were calculated using Equations (1) and (2), respectively. In these equations, Y grain is seed yield (kg ha^−1^), B total is total aboveground biomass at maturity (kg ha^−1^), NA grain is nutrient accumulation (N, P, or K) in grain, and NA total is total aboveground nutrient accumulation.

(1) HI (%) = (Y grain/B total) × 100%

(2) NHI (%) = (NA grain/NA total) × 100%

Agronomic efficiency (AE), recovery efficiency (RE), and partial factor productivity (PFP) were calculated using Equations (3)–(5). In these equations, Y_T_ and Y_CK_ are grain yields in fertilized and unfertilized (CK) plots, respectively; U_T_ and U_CK_ are total nutrient uptake in fertilized and unfertilized plots, respectively; and F is the amount of applied fertilizer nutrient. These indices were calculated separately for N, P, and K using the corresponding nutrient uptake and application rates. In addition, the proportion of total N, P, or K present in available forms was calculated as AC (%) = (Nutrient available/Nutrient total) × 100, where Nutrient available is the concentration of available N, P, or K (mg kg^−1^), and Nutrient total is the concentration of total N, P, or K in the 0–20 cm soil layer after conversion from g kg^−1^ to mg kg^−1^.

(3) AE = (Y_T_ − Y_CK_)/F

(4) RE (%) = 100 × (U_T_ − U_CK_)/F

(5) PFP = Y_T_/F

(6) AC (%) = (Nutrient_available_/Nutrient_total_) × 100%

For structural equation modeling (SEM), fertilizer source structure and several composite functional indices were constructed to represent coordinated variable modules and to reduce collinearity among closely related variables. Organic substitution rate (S) was defined as the percentage of mineral N replaced by composted manure in each fertilized treatment, with F = 0, 25%M = 25, 50%M = 50, 75%M = 75, and 100%M = 100. Before index construction, all variables were z-standardized across fertilized plot-year observations so that variables measured in different units contributed on a comparable scale and path coefficients could be directly compared [49]. The Enzyme index was calculated as the mean of the standardized values of β-GC, CBH, LAP, NAG, and ALP; the Soil Pool index as the mean of SOC, TN, TP, and TK; the Available index as the mean of AN, AP, and AK; the Uptake index as the mean of plant N, P, and K uptake; and the RE index as the mean of NRE, PRE, and KRE. The SEM was fitted using plot-year observations from fertilized treatments only (F and 25%M–100%M), because S is not defined for the unfertilized control. Piecewise SEM was used because it allows the causal network to be estimated as a set of component models and is suitable for data structures with hierarchical or repeated observations [50,51]. Standardized path coefficients (β) were reported to facilitate comparison of path strength within the model. Model fit was evaluated using tests of directed separation and Fisher’s C, with a non-significant Fisher’s C test (*p* > 0.05) indicating that the hypothesized causal structure was broadly consistent with the data [50,51].

Economic analysis of Brassica napus production was performed using a partial budget approach. Net income (NI) was calculated as NI = TR − (FC + VC), where TR is total return, calculated as seed yield multiplied by the local market price; FC is fixed cost, including seeds, plant protection products (herbicides, insecticides, and fungicides), labor, and machinery operations (plowing, sowing, and harvesting); and VC is variable cost, consisting mainly of expenditures on chemical fertilizers and organic amendments.

(7) NI = TR − (FC + VC)

### 4.7. Statistical Analysis

Data are presented as mean ± SE (*n* = 3). For each year, treatment effects were tested by analysis of variance (ANOVA) for a randomized complete block design, with treatment as a fixed effect and block as a blocking factor. When the treatment effect was significant, means were compared using Fisher’s LSD test at *p* < 0.05. These analyses were used for within-year treatment comparisons and for the lowercase letter groupings shown in the figures.

To assess cross-year variation in treatment responses, the combined two-year dataset was analyzed by two-way ANOVA with Year, Treatment, and their interaction (Year × Treatment) as fixed effects. This analysis was used to evaluate the overall Year effect shown in the figures. Fertilizer-use efficiency indices were analyzed using fertilized treatments only, because they are not defined for the unfertilized control (CK). Pearson correlations were calculated separately for each year using fertilized plots only and visualized as heatmaps. The piecewise SEM was fitted using fertilized plot-year observations only, as described in Section 4.6. When necessary, data were log- or square-root transformed to improve normality and homogeneity of variance. All analyses were performed in R (version 4.5.2), and figures were prepared in Origin 2024. Differences were considered significant at *p* < 0.05.

## 5. Conclusions

This two-year field study showed that an intermediate manure-mineral fertilizer strategy provided the most balanced overall performance for alpine rapeseed grown under cold, short-season conditions. Among the tested treatments, 50% substitution of mineral N with organic fertilizer produced the highest grain yield and the strongest overall nutrient-use efficiency, together with greater soil enzyme activity, higher nutrient availability, and improved plant nutrient uptake and recovery. By contrast, full substitution increased SOC and total nutrient pools but did not maintain comparable in-season nutrient availability, resulting in lower recovery efficiency and a slight yield penalty. The SEM further indicated that yield responses were more closely associated with nutrient availability, uptake, and recovery than with expansion of total soil nutrient pools alone. Overall, these results support partial rather than complete substitution of mineral fertilizer with organic fertilizer, with a substitution proportion around 50% appearing most suitable under the tested alpine conditions.

## Figures and Tables

**Table 1:** Baseline physicochemical properties of the topsoil (0–20 cm) prior to the experiment in 2024.

SOC (g kg^−1^)	TN (g kg^−1^)	TP (g kg^−1^)	TK (g kg^−1^)	AN (mg kg^−1^)	AP (mg kg^−1^)	AK (mg kg^−1^)	pH
12.35 ± 0.09	1.08 ± 0.06	0.74 ± 0.05	18.1 ± 0.3	69.6 ± 3.7	10.3 ± 0.7	115.8 ± 8.4	8.2 ± 0.1

Note: Values are presented as mean ± standard deviation (*n* = 3).

**Table 2:** Experimental design and fertilizer application rates (dry-matter basis) for different treatments.

Treatment	Chemical Fertilizers			Organic Fertilizer			Organic Fertilizer (kg·ha^−1^ Dry Matter)
	N (kg·ha^−1^)	P_2_O_5_ (kg·ha^−1^)	K_2_O (kg·ha^−1^)	N (kg·ha^−1^)	P_2_O_5_ (kg·ha^−1^)	K_2_O (kg·ha^−1^)	
CK	0	0	0	0	0	0	0
F	105.00	105.00	45.00	0	0	0	0
25%M	78.75	82.99	35.57	26.25	22.01	9.43	1367.19
50%M	52.50	60.98	26.13	52.50	44.02	18.87	2734.38
75%M	26.25	38.97	16.70	78.75	66.03	28.30	4101.56
100%M	0	16.95	7.27	105.00	88.05	37.73	5468.75

Note: CK, control (no fertilizer); F, 100% chemical fertilizer (farmers’ practice); 25%M, 50%M, 75%M, and 100%M represent the substitution of chemical N with composted manure at rates of 25%, 50%, 75%, and 100%, respectively. Manure application rates are reported as dry-matter (DM)-equivalent amounts; manure was applied in the field as received, and the DM-equivalent rates were calculated using the year-specific manure moisture content and nutrient concentrations. The total inputs of N, P_2_O_5_, and K_2_O were kept consistent within each year across all fertilized treatments.

## Data Availability

The original contributions presented in this study are included in the article. Further inquiries can be directed to the corresponding author.

## Abbreviations

The following abbreviations are used in this manuscript:

AE	Agronomic efficiency
AEK	Agronomic efficiency of potassium
AEN	Agronomic efficiency of nitrogen
AEP	Agronomic efficiency of phosphorus
AK	Available potassium
ALP	Alkaline phosphatase
AN	Available nitrogen
AP	Available phosphorus
BBCH	Biologische Bundesanstalt, Bundessortenamt and CHemical industry growth scale
CBH	Cellobiohydrolase
CK	Control (no fertilizer)
CNY	Chinese yuan
DM	Dry matter
F	100% mineral fertilizer
HI	Harvest index
K_2_O	Potassium oxide
KAC	Potassium availability coefficient
KUE	Potassium use efficiency
LAP	Leucine aminopeptidase
N	Nitrogen
NAC	Nitrogen availability coefficient
NAG	N-acetyl-β-D-glucosaminidase
NHI	Nutrient harvest index
NHI-K	Potassium harvest index
NHI-N	Nitrogen harvest index
NHI-P	Phosphorus harvest index
NI	Net income
NUE	Nitrogen use efficiency
P	Phosphorus
P_2_O_5_	Phosphorus pentoxide
PAC	Phosphorus availability coefficient
PFP	Partial factor productivity
PFPK	Partial factor productivity of potassium
PFPN	Partial factor productivity of nitrogen
PFPP	Partial factor productivity of phosphorus
PUE	Phosphorus use efficiency
RE	Recovery efficiency
REK	Recovery efficiency of potassium
REN	Recovery efficiency of nitrogen
REP	Recovery efficiency of phosphorus
SEM	Structural equation model
SOC	Soil organic carbon
TK	Total potassium
TN	Total nitrogen
TP	Total phosphorus
β-GC	β-glucosidase
